# Effects of Sample Size on Differential Gene Expression, Rank Order and Prediction Accuracy of a Gene Signature

**DOI:** 10.1371/journal.pone.0065380

**Published:** 2013-06-03

**Authors:** Cynthia Stretch, Sheehan Khan, Nasimeh Asgarian, Roman Eisner, Saman Vaisipour, Sambasivarao Damaraju, Kathryn Graham, Oliver F. Bathe, Helen Steed, Russell Greiner, Vickie E. Baracos

**Affiliations:** 1 Department of Oncology, University of Alberta, Cross Cancer Institute, Edmonton, Alberta, Canada; 2 Department of Computing Science, University of Alberta, Edmonton, AB, Canada; 3 Department of Laboratory Medicine and Pathology, University of Alberta, Edmonton, AB, Canada; 4 Department of Oncology, University of Calgary, Calgary, Alberta, Canada; 5 Department of Surgery, University of Calgary, Calgary, Alberta, Canada; 6 Alberta Innovates Centre for Machine Learning, Edmonton, AB, Canada; National Institute of Environmental Health Sciences, United States of America

## Abstract

Top differentially expressed gene lists are often inconsistent between studies and it has been suggested that small sample sizes contribute to lack of reproducibility and poor prediction accuracy in discriminative models. We considered sex differences (69♂, 65♀) in 134 human skeletal muscle biopsies using DNA microarray. The full dataset and subsamples (n = 10 (5♂, 5♀) to n = 120 (60♂, 60♀)) thereof were used to assess the effect of sample size on the differential expression of single genes, gene rank order and prediction accuracy. Using our full dataset (n = 134), we identified 717 differentially expressed transcripts (p<0.0001) and we were able predict sex with ∼90% accuracy, both within our dataset and on external datasets. Both p-values and rank order of top differentially expressed genes became more variable using smaller subsamples. For example, at n = 10 (5♂, 5♀), no gene was considered differentially expressed at p<0.0001 and prediction accuracy was ∼50% (no better than chance). We found that sample size clearly affects microarray analysis results; small sample sizes result in unstable gene lists and poor prediction accuracy. We anticipate this will apply to other phenotypes, in addition to sex.

## Introduction

Microarray technology has been adopted to gain a comprehensive picture of gene expression differences. In human studies, the sample size is often limited because microarray technology is quite costly and the required tissue biopsies may be invasive. For example, in the quest to understand sexual dimorphism in human skeletal muscle gene expression, the early report by Roth et al. [1] studied pooled samples from 5 men and 5 women on 4K arrays (Invitrogen). Later, several other groups studied samples from 6 to 15 subjects per sex on 45K arrays (Affimetrix) [2]–[4]. Such sample sizes are not unusual in gene array studies on human tissues [5].

A lack of concordance is evident in the gene lists generated in studies that compared the same phenotypes. For example, amongst the top 20–30 differentially expressed genes reported in the two studies cited above (by Welle et al. and by Maher et al.), only 5 were common to both lists: ALDH4A1, DAAM2, INSR, IRX3, TPD52. The issue of poor overlap of gene lists across studies has raised doubts about their reliability and robustness of gene signatures in general [6].

Microarray studies are conducted either: (1) to identify differentially expressed genes between groups (e.g. towards understanding underlying biological mechanisms) and/or (2) to identify patterns of gene expression that can be used to develop a predictor with high accuracy (e.g. for diagnosis of a disease) [7]. Researchers typically report the top differentially expressed genes and these are often credited with high importance, however the reproducibility of the identity and rank order (i.e. 1^st^ or 50^th^ most differentially expressed) is usually not addressed.

Sample size is proposed to be an important determinant of the number of differentially expressed genes reliably detected as well as the accuracy of a predictor [8]–[12]. Some prior studies have considered what sample size is required to ensure that the genes associated with a phenotype can be discovered with a minimal false discovery rate [13]; others explore the effect of sample size on the overlap of gene lists [8], [9]; and yet others have investigated the effect of sample size on the likelihood of identifying true associations among the top ranked genes [14]. In general, these analyses consider various sub-samples of a given large initial dataset, to determine how well each size of subsamples approximates the findings made using the entire dataset. Because of a general paucity of large datasets, authors either used computer-simulated datasets [8], [9], or created data pools by combining independent datasets [8], [15]. However, simulated data does not necessarily reflect biological variation and pooling of data from different studies by different investigators introduces batch effects and thereby increase variability [5], [16]. We can avoid these problems by using a single large dataset acquired on the same platform, lab and experimental condition. It is also important that the class label (phenotype) be unambiguous. An objective class label (e.g. male vs. female) rather than subjective (e.g. estrogen receptor status, subject to measurement error and based on the subjective opinion of an individual pathologist [17]) would be ideal. A subjective class label may contaminate the dataset with incorrectly labeled instances and therefore introduce variation.

Here, we used sexual dimorphism in human skeletal muscle gene expression using a single large (n = 134) dataset with 41K Agilent arrays, as a model to assess the effect of sample size on the differential expression, rank order and prediction tasks. For the association analyses, our goal was to determine the consistency of the rank orderings of the genes, from one size-n sample to another; this is different from other studies that attempt to determine how many of the top biomarkers are “correct” [14].

## Materials and Methods

### Ethics Statement

This study was approved by the Alberta Cancer Research Ethics Committee. Patients provided written consent. Tissues were stored at the Alberta Cancer Research Biorepository/Canadian Breast Cancer Foundation Tumor Bank and the University of Calgary HPB/GI Tumor Tissue Bank.

### Subjects and Acquisition of Muscle Samples

Adult (>18 yrs) cancer patients underwent open abdominal surgery as part of their clinical care. Biopsies of *rectus abdominis* muscle (0.5–1 g) were taken from the site of incision, at the start of surgery using sharp dissection and without the use of electrocautery. Biopsies were immediately frozen in liquid nitrogen and stored in liquid nitrogen vapor phase until analysis. Skeletal muscle index (cm^2^/m^2^), whole body skeletal muscle estimation (kg) and rate of muscle change (% change/100d) was assessed from computed tomography images taken prior to biopsy as part of routine clinical care as described previously [18]–[20]. Age and cancer type were abstracted from medical charts.

### Microarray Analysis

Total RNA was isolated using Trizol (Sigma-Aldrich, Oakville, ON, CAN), purified using Qiagen RNeasy columns (Mississauga, ON, CAN), quantified using a NanoDrop 1000 Spectrophotometer (NanoDrop Technologies, Wilmington, DE, USA) and its integrity evaluated using a Bioanalyzer 2100 (Agilent Technologies, Santa Clara, CA, USA) according to manufacturer’s protocols. All RNA samples had RNA Integrity Numbers (RIN) greater than 7.0.

RNA was subjected to linear amplification and Cy3 labeling and hybridization to Agilent Whole Human Genome Arrays using Agilent kits (One Color Low RNA Input Linear Amplification Kit Plus, One Color RNA Spike-In Kit and Gene Expression Hybridization Kit). The arrays were scanned using an Agilent Scanner, the data was extracted and quality was evaluated using Feature Extraction Software 10.5.1 (Agilent). The data was normalized using GeneSpring GX 11.5.1 (Agilent). The data used in this publication have been deposited in the U.S. National Center for Biotechnology Information (NCBI) Gene Expression Omnibus25 and are accessible through GEO series accession number GSE41726.

### Statistical Analysis

There were a total of 41,000 oligonucleotide sequences (i.e. transcripts) on each microarray chip. This produces a dataset that describes each of 134 subjects (69 men and 65 women), using 41,000 transcripts (each a real number) and sex (either M or F). Microarray intensity values were log transformed prior to analyses.

#### Effect of sample size on differentially expressed gene lists

For each sample size considered (n = 10 (5♀, 5♂), 20 (10♀, 10♂), … 120 (60♀, 60♂)), we randomly selected a size-n subsample (containing equal numbers of men and women) from our dataset of n = 134. For each of these size-n subsamples, we computed the t-test on the (log transformed) intensities over the set of males vs. the set of females. We repeated this procedure 50 times for each sample size n and then for each gene, averaged the p-values computed over these 50 trials. Mean p-values were then sorted from lowest to highest to determine top 100 transcripts for each sample size. We also evaluated how the specific rank order of top genes was affected by sample size. For each size n subsample we assigned a rank value (1 to 100) to each gene, based on its p-value. We then sorted the gene based on its mean rank (for each sample size), based on all 50 repeats. As our main focus was this ranking, we simply used the p-values from the t-tests, rather than any multiplicity-corrected variant (such as the Benjamini-Hogeberg correction [21]). If we had used a multiplicity correction, enforced monotonicity would have been required to ensure the ranking of adjusted p-values remain unchanged. A method of enforced monotinicity was presented by Yekutieli and Benjamini [22].

#### Effect of sample size on prediction accuracy

As XY chromosome transcripts (1,548 transcripts) are obviously highly related to sex, a single XY transcript may be sufficient to build a classifier that could predict sex perfectly. To generate a more typically physiological prediction problem we therefore excluded these transcripts when building classifiers. We used the LASSO algorithm (implemented using R, glmnet package) [23]. Given a training dataset, LASSO produces a classifier that predicts the class label (sex) of a new patient from his/her microarray data. In general, the quality of a classifier is its predictive accuracy (% correct classification) on novel subjects; we used 10-fold cross validation to internally validate the model. To determine how sample size of the training dataset affects sex prediction accuracy, we trained classifiers using randomly selected sub-samples of our data (n = 10 (5♀, 5♂) to n = 110 (55♀, 55♂)). We repeated this 50 times for each n.

To externally validate our model, we used publicly available datasets that used the same tissue (i.e. skeletal muscle) and platform (i.e. Agilent), for which the sex was known: dataset GSE24215 included microarray data from 10 healthy, young men and dataset GSE23697 included 34 healthy, adult men. To determine how sample size of the training dataset affects sex prediction accuracy on these external datasets, we trained classifiers using randomly selected subsamples of our data (n = 10 (5♀, 5♂) to n = 110 (55♀, 55♂)) then used these learned classifiers to predict sex on the external datasets. We repeated this 50 times for each n.

## Results

Gene expression microarray analysis was conducted on 134 *rectus abdominus* muscle biopsies (69♂, 65♀). The characteristics of the study participants are shown (Table 1). As expected, men were 26% more muscular than women (t-test, p<0.0001). Mean age and number of patients undergoing chemotherapy did not differ between the men and women in this study.

**Table 1:** Patient characteristics.

	Men	Women
**Total, n**	69	65
**Age, mean years ± SD**	59±13	63±13
**Muscle, mean ± SD**		
Skeletal muscle index (cm^2^/m^2^)	52.9±7.8	41.9±8.3*
^ 1^Estimated whole body skeletal muscle, kg	27.0±4.8	17.9±3.7*
Muscle rate of change, %/100d	−4.4±10.9	−4.5±12.5
**Diagnosis at surgery, %**		
Benign neoplasm	13	18
Cancer, liver or intrahepatic bile ducts	17	14
Cancer, gastrointestinal tract	46	22
Cancer, pancreas	19	25
Cancer, ovary or uterus	0	17
Cancer, head and neck	3	2
Cancer, skin	0	2
Cancer, kidney	1	0

* Different from men, p-value <0.0001.

1 Derived regression equations [19].

### Effect of Sample Size on Differential Expression

The full dataset was checked for differential gene expression revealed 717 differentially expressed transcripts with a p-value <0.0001. Note that the biological interpretation of these differentially expressed genes is not the goal of this study, and so will be discussed in a separate work.

**Figure 1:**
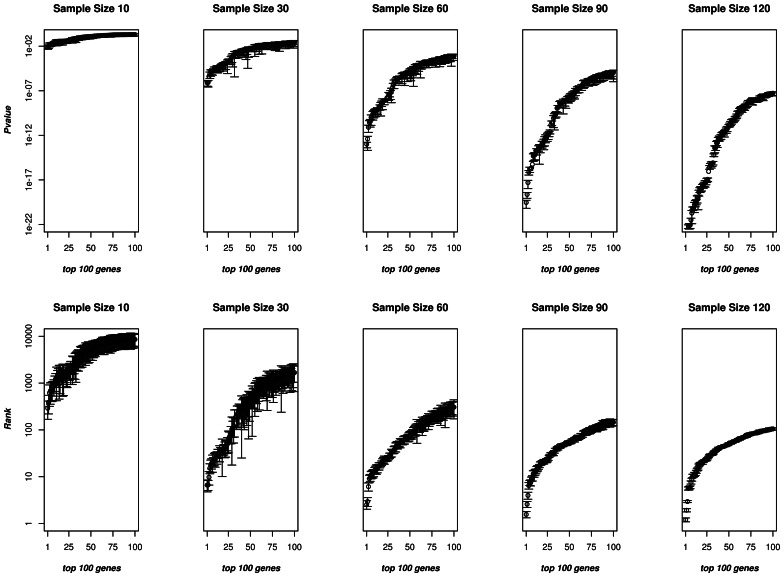
The effect of sample size on p-values and rank order for the top 100 transcripts. For each n tested (n = 10 (5♀, 5♂), 30 (15♀, 15♂), 60 (30♀, 30♂), 90 (45♀, 45♂) and 120 (60♀, 60♂) are shown here), n samples were randomly selected from our dataset of n = 134 subjects, 50 different times. Top panels: The average and 95% confidence intervals of the p-values for the top 100 transcripts. As sample size was increased, the average p-value decreased and became less variable. Bottom panels: The average and 95% confidence intervals rank for the top 100 transcripts. As sample size was increased, the average rank decreased and became less variable.

This analysis was repeated for random samples of n = 10 (5♀, 5♂) to n = 120 (60♀, 60♂) increasing the sample size by increments of 5♀ and 5♂ (Figure 1, top panels). At n = 10 (5♀, 5♂), no genes were significant at p-value <0.0001 whereas at n = 120 (60♀, 60♂), there were 472 differentially expressed transcripts at the same p-value cutoff. Of course, the variance of these measurements become less meaningful for large subsamples, as the different size-n subsamples will have high overlap since they are all drawn from our dataset of 134 (65♀, 69♂). The variances, however, are fairly accurate for small values of n. To assess the similarity of sample sets, we calculated the median Jaccard score over 1000 randomly generated pairs of subsamples of size n. The Jaccard score of two sets A and B is the size of the intersection divided by the size of the union, i.e. J(A,B) = |A ∩B|/|A∪B|. Note that the Jaccard score is always between 0 and 1; the score of 0 means the two sets are disjoint, while the score of 1 means they are identical. As the median Jaccard score for two n = 30 (15♀, 15♂) subsamples is around 0.1, the overlap is very small. Such sizes are the most relevant, as they reflect the sizes of many earlier human microarray studies. Below we consider n = 60 (30♀, 30♂); we consider the observed variations relevant as the Jaccard scores here are still under 0.3.

We then explored whether the ranking of the genes were reproducible over different sample sizes. From the previous analysis, for each of the 50 random samplings, each gene was given a ranking based on the p-value of its t-test (e.g. if a gene is ranked 4^th^, or 25^th^, or 120^th^), see Figure 1, bottom panels. In Figure 1 (bottom right-hand panel), in a large sample (n = 60 (30♀, 30♂)) the top three genes (PRKY, DDX3Y, UTY) were reproducibly identified in the top 3 ranks in all 50 iterations of sampling. By contrast, the p-value of 10^th^ ranked gene was very close to its immediate neighbors; while on average it ranked 10^th^, its rank ranged from 5^th^ to 17^th^. As we decreased n, the ranking of any given gene became more and more variable, in that the rank of every gene had a larger range (e.g. at sample size n = 30 (15♀, 15♂), the gene whose average rank was 10^th^, ranged in rank from 1^st^ to 127^th^ in the different random subsamples).

### Effect of Sample Size on Prediction Accuracy

Microarray data are sometimes used to make a prediction (i.e. to determine the phenotype of a future subject (e.g. healthy or disease), based on a classifier produced from prior subjects. While there is no clinical need to predict a person’s sex using muscle gene expression array, our data does provide the opportunity to explore the relationship between n and the ability to build a robust predictor. The classifier based on all n = 134 subjects used only 92 genes of the complete set of 41,000 and could predict sex with mean 92.5±7.3% (10-fold) cross-validation accuracy. We then explored the predictive accuracy of this model on publicly available muscle expression array data obtained in unrelated investigations conducted on the same platform (Agilent). In two such external datasets, this model had excellent accuracy: correctly predicted sex for 9/10 subjects and for 35/35 subjects in dataset GSE24215 and dataset GSE23697, respectively.

**Figure 2:**
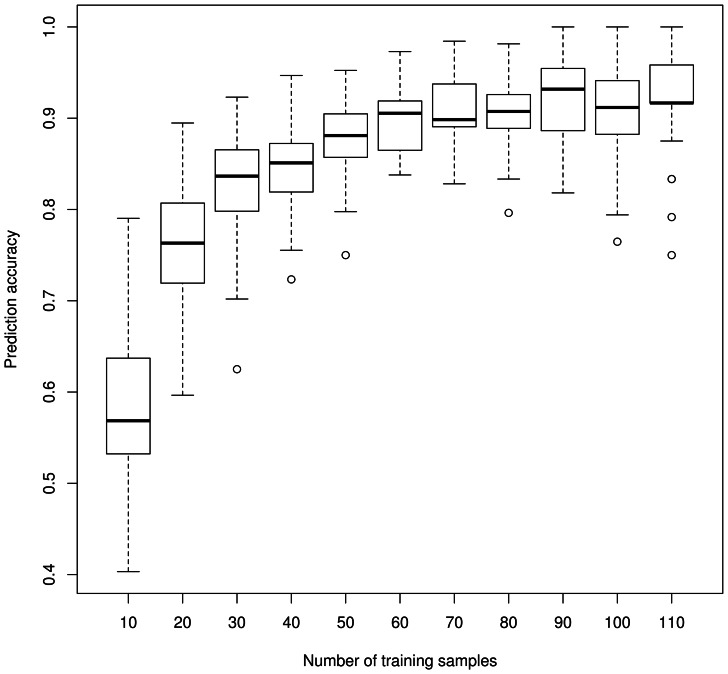
Box-and-whiskers plot showing the mean internal cross-validation accuracy of sex prediction for different sample sizes. Sample sizes tested ranged from n = 10 (5♀, 5♂) to n = 110 (55♀, 55♂). To calculate the mean 10-fold cross validation prediction accuracy, for each n ( = 10…110), we built classification models using a randomly selected size-n subsamples of our full dataset of n = 134. This was repeated 50 times and the median prediction accuracy for each sample was calculated. As sample size increased, so did prediction accuracy.

**Figure 3:**
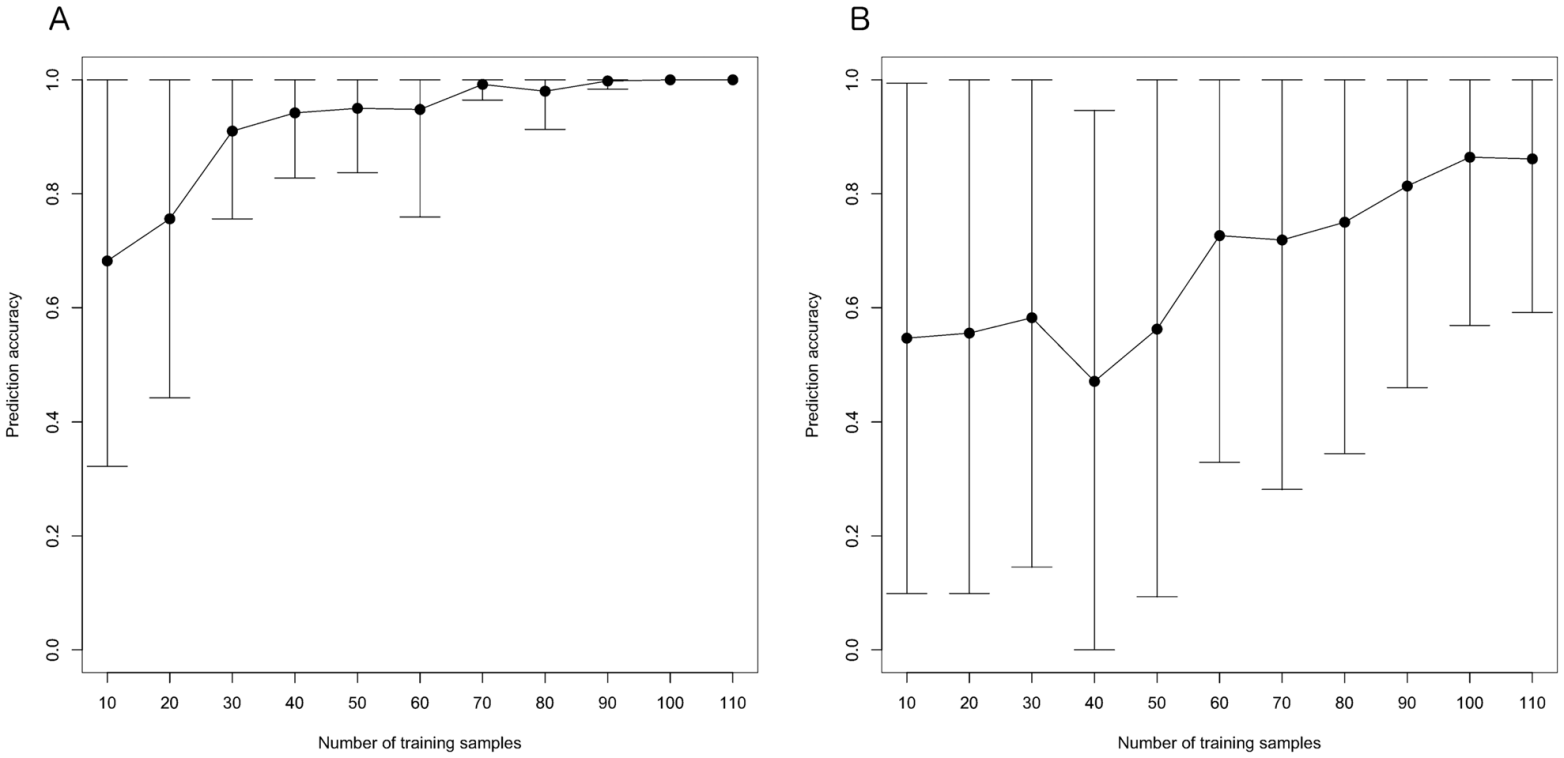
Plots showing the mean and standard deviation accuracy of sex prediction on two external datasets using a predictor trained using different sample sizes from our dataset. We built predictors using different training sample sizes ranging from n = 10 (5♀, 5♂) to n = 110 (55♀, 55♂) from our full dataset. We then calculated the prediction accuracy, for each n ( = 10…110) on two external datasets (A. Dataset GSE24215 and B. Dataset GSE23697). This was repeated 50 times and the mean and standard deviation prediction accuracy for each sample size was calculated. As the training sample size increased, so did prediction accuracy on the external datasets.

Figure 2 shows the mean internal cross-validation accuracy of sex prediction as we varied the sample size of the training data from n = 10 (5♀, 5♂) to n = 110 (55♀, 55♂). When the training sample had n = 10 (5♀, 5♂), the classifier was unable to predict sex any better than chance (∼50% cross validation accuracy). This accuracy increased as we increased n; we achieved predictive accuracy above 90% when training on a sample of at least 80 individuals (40 of each sex). This trend of increased accuracy with increased n was also seen when we used different subsamples of size n = 10 (5♀, 5♂) to n = 110 (55♀, 55♂) from our dataset to predict sex on the external datasets mentioned above (Figure 3).

## Discussion

Our empirical evidence suggests that small sample sizes often typical of microarray studies negatively affect their interpretation, whether used to determine differential gene expression or to accurately predict future instances. The relatively high cost of analyses and the invasiveness of sampling tissues such as skeletal muscle in humans often dictate rather small sample sizes [24], [25] but our results suggest that efforts to increase n may well be justified.

Researchers in biology attribute great importance to top ranked gene(s) in differential expression analyses [26]. This is the first study examining the effect of sample size on gene rank using one large dataset and a biologically unambiguous label. We show that any given gene may have a wide range of ranks, especially for small sample sizes. For example, in 50 subsamples of size n = 20 (10♀, 10♂), the gene that had the highest average rank, sometimes appeared in rank 200. By contrast, at n = 60 (30♀, 30♂) the top three ranked genes were constant. These observations explain the lack of concordance between the findings of two prior studies of sexual dimorphism [2], [4] with each other, and with our results. Those two earlier studies had 6 to 15 of each sex in their analysis and the 5 genes that they had in common with each other did not all rank in our top 100 differentially expressed gene list (these genes ranked 49^th^ (IRX3), 62^nd^ (DAAM2), 67^th^ (TPD52), 147^th^ (ALDH4A1) or was not significant (p = 0.3, rank = 18854) (INSR) at n = 134 (our full dataset).

Microarray analysis is often used to identify gene signatures that can be used to develop a predictor. In agreement with previous studies looking at this methodological issue, we conclude that small sample sizes (e.g. n <20 per class label) will often result in poor predictors [8], [10], [15], [27], but the accuracy improves with increased sample size in the training dataset. This was the case both within our data and, as shown in Figures 3, when trying to make predictions on external datasets. In our study, a LASSO predictor, trained on our full dataset (n = 134) returned 90–100% accuracy on publicly available data (external validation). This excellent predictive accuracy also suggests that our findings of sex-related gene expression are not confounded by the use of a cancer patient sample, because the predictor based on these patients was accurate on data obtained on healthy men and women.

Sample size is not the only factor that can influence microarray analysis. Indeed, incomplete annotation of the genome and probes targeted to different regions of the encoding gene, stringency of hybridization conditions, commercially available arrays vs. in-house built, pre-analytical variables in the tissue accrual including induced hypoxia concomitant post de-vitalization of tissue and temperature and duration of storage of tissues should also be considered when comparing previous or designing future microarray experiments. Here, we focused on sample size while maintaining the tissue collection method, microarray platform and storage conditions constant for all samples. Our analysis suggests principles that dictate how ranking and prediction accuracy can vary, in relation to the biological label (sex) that we chose to study. Studies with larger inherent variance in the data (e.g. due to batch effects introduced by pooling several datasets) may require considerably larger sample sizes than we report here [8]–[10], [28]. By contrast in animal experiments which permit extensive control of many sources of variation, smaller sample sizes may be sufficient to test similar experimental questions. Thus, it is not possible to state how many genes will be reliable/reproducible at different sample sizes for other datasets *a priori*. However, it would be beneficial to assess how sample size may affect ranking and prediction tasks, as we did here, by examining the robustness of top-ranked genes and mean and variance of cross-validated results for different subsamples of varying n, respectively. Even if a dataset is deemed to have a sufficient sample size, there are other methodological considerations that were not addressed here but which are important to properly interpret the data. For example, researchers need to carefully consider what multiplicity correction should be used, which depends on the properties of the dataset in questions (e.g. the normality of the data).

We conclude that gene signatures generated from small datasets should be interpreted with caution as they may not be reproducible and that prediction models built using small sample sizes result in poor prediction accuracy. While we cannot recommend specific sample sizes, outside the problem that we studied, our analysis shows that the sample size n = 10 (5♀, 5♂) was not useful for either prediction (which was not better than chance) nor for association (the probability of finding reproducible top 10 genes was negligible).

### For More Information

Links to datasets used for external validation of sex prediction model:

Dataset GSE24215: http://www.ncbi.nlm.nih.gov/projects/geo/query/acc.cgi?acc=GSE24215.

Dataset GSE23697: http://www.ncbi.nlm.nih.gov/projects/geo/query/acc.cgi?acc=GSE23697.

## References

[1] Roth SM, Ferrell RE, Peters DG, Metter EJ, Hurley BF, et al (2002) Influence of age, sex, and strength training on human muscle gene expression determined by microarray. Physiol Genomics 10: 181–190. 12209020 10.1152/physiolgenomics.00028.2002 PMC2812433

[2] Welle S, Tawil R, Thornton CA (2008) Sex-related differences in gene expression in human skeletal muscle. PLoS One 3: e1385. 18167544 10.1371/journal.pone.0001385 PMC2148100

[3] Liu D, Sartor MA, Nader GA, Gutmann L, Treutelaar MK, et al (2010) Skeletal muscle gene expression in response to resistance exercise: sex specific regulation. BMC Genomics 11: 659. 21106073 10.1186/1471-2164-11-659 PMC3091777

[4] Maher AC, Fu MH, Isfort RJ, Varbanov AR, Qu XA, et al (2009) Sex differences in global mRNA content of human skeletal muscle. PLoS One 4: e6335. 19623254 10.1371/journal.pone.0006335 PMC2709437

[5] Klebanov L, Yakovlev A (2007) Is there an alternative to increasing the sample size in microarray studies? Bioinformation 1: 429–431. 17597934 10.6026/97320630001429 PMC1896058

[6] Michiels S, Koscielny S, Hill C (2005) Prediction of cancer outcome with microarrays: a multiple random validation strategy. Lancet 365: 488–492. 15705458 10.1016/S0140-6736(05)17866-0

[7] Simon R (2003) Diagnostic and prognostic prediction using gene expression profiles in high-dimensional microarray data. Br J Cancer 89: 1599–1604. 14583755 10.1038/sj.bjc.6601326 PMC2394420

[8] Kim SY (2009) Effects of sample size on robustness and prediction accuracy of a prognostic gene signature. BMC Bioinformatics 10: 147. 19445687 10.1186/1471-2105-10-147 PMC2689196

[9] Ein-Dor L, Zuk O, Domany E (2006) Thousands of samples are needed to generate a robust gene list for predicting outcome in cancer. Proc Natl Acad Sci U S A 103: 5923–5928. 16585533 10.1073/pnas.0601231103 PMC1458674

[10] van Vliet MH, Reyal F, Horlings HM, van de Vijver MJ, Reinders MJ, et al (2008) Pooling breast cancer datasets has a synergetic effect on classification performance and improves signature stability. BMC Genomics 9: 375. 18684329 10.1186/1471-2164-9-375 PMC2527336

[11] Ramasamy A, Mondry A, Holmes CC, Altman DG (2008) Key issues in conducting a meta-analysis of gene expression microarray datasets. PLoS Med 5: e184. 18767902 10.1371/journal.pmed.0050184 PMC2528050

[12] Pusztai L, Mazouni C, Anderson K, Wu Y, Symmans WF (2006) Molecular classification of breast cancer: limitations and potential. Oncologist 11: 868–877. 16951390 10.1634/theoncologist.11-8-868

[13] Matsui S, Oura T (2009) Sample sizes for a robust ranking and selection of genes in microarray experiments. Stat Med 28: 2801–2816. 19610132 10.1002/sim.3666

[14] Kuo CL, Zaykin DV (2011) Novel rank-based approaches for discovery and replication in genome-wide association studies. Genetics 189: 329–340. 21705758 10.1534/genetics.111.130542 PMC3176128

[15] Dobbin KK, Zhao Y, Simon RM (2008) How large a training set is needed to develop a classifier for microarray data? Clin Cancer Res 14: 108–114. 18172259 10.1158/1078-0432.CCR-07-0443

[16] Bolstad BM, Collin F, Simpson KM, Irizarry RA, Speed TP (2004) Experimental design and low-level analysis of microarray data. Int Rev Neurobiol 60: 25–58. 15474586 10.1016/S0074-7742(04)60002-X

[17] Diaz LK, Sneige N (2005) Estrogen receptor analysis for breast cancer: current issues and keys to increasing testing accuracy. Adv Anat Pathol 12: 10–19. 15614160 10.1097/00125480-200501000-00003

[18] Eisner R, Stretch C, Eastman T, Xia J, Hau D, et al (2011) Learning to predict cancer-associated skeletal muscle wasting from 1H-NMR profiles of urinary metabolites. Metabolomics 7: 25–34.

[19] Shen W, Punyanitya M, Wang Z, Gallagher D, St-Onge MP, et al (2004) Total body skeletal muscle and adipose tissue volumes: estimation from a single abdominal cross-sectional image. J Appl Physiol 97: 2333–2338. 15310748 10.1152/japplphysiol.00744.2004

[20] Murphy RA, Mourtzakis M, Chu QS, Baracos VE, Reiman T, et al (2011) Nutritional intervention with fish oil provides a benefit over standard of care for weight and skeletal muscle mass in patients with nonsmall cell lung cancer receiving chemotherapy. Cancer 117: 1775–1782. 21360698 10.1002/cncr.25709

[21] Benjamini Y, Hogchberg Y (1995) Controlling the False Discovery Rate: a practical and powerful approach to multiple testing. J Royal Stat Soc Ser B 1: 289–300.

[22] Yekutieli D BY (1999) Resampling-based false discovery rate controlling multiple test procedures for correlated test statistics. Journal of Statistical Planning and Inference 82: 171–196.

[23] Tibshirani R (1996) Regression shrinkage and selection via the lasso. J R Statist Soc B 58: 267–288.

[24] Timmons JA, Sundberg CJ (2006) Oligonucleotide microarray expression profiling: human skeletal muscle phenotype and aerobic exercise training. IUBMB Life 58: 15–24. 16540428 10.1080/15216540500507390

[25] Virtanen C, Takahashi M (2008) Muscling in on microarrays. Appl Physiol Nutr Metab 33: 124–129. 18347662 10.1139/H07-150

[26] Fluck M, Dapp C, Schmutz S, Wit E, Hoppeler H (2005) Transcriptional profiling of tissue plasticity: role of shifts in gene expression and technical limitations. J Appl Physiol 99: 397–413. 16020435 10.1152/japplphysiol.00050.2005

[27] Popovici V, Chen W, Gallas BG, Hatzis C, Shi W, et al (2010) Effect of training-sample size and classification difficulty on the accuracy of genomic predictors. Breast Cancer Res 12: R5. 20064235 10.1186/bcr2468 PMC2880423

[28] Damavandi B (2012) Estimating the overlap of top instances in lists ranked by correlation to label. Edmonton, Alberta: University of Alberta. 52 p.

